# Preparation of milk‐based probiotic lactic acid bacteria biofilms: A new generation of probiotics

**DOI:** 10.1002/fsn3.3273

**Published:** 2023-02-22

**Authors:** Zeinab Rezaei, Amir Salari, Saeid Khanzadi, Jong‐Whan Rhim, Ehsan Shamloo

**Affiliations:** ^1^ Department of Food Hygiene and Aquaculture, Faculty of Veterinary Medicine Ferdowsi University of Mashhad Mashhad Iran; ^2^ Department of Food and Nutrition, BioNanocomposite Research Center Kyung Hee University Seoul Republic of Korea; ^3^ Department of Food Science and Technology Neyshabur University of Medical Sciences Neyshabur Iran

**Keywords:** biofilm, *Lacticaseibacillus rhamnosus*, *Lactiplantibacillus plantarum*, new generation of probiotics, probiotics, yogurt

## Abstract

Biofilm is considered as a community of microorganisms in which cells adhere to each other on surfaces in a self‐produced matrix of extracellular polymer compounds. In recent years, efforts to use the beneficial aspects of biofilm in probiotic research have intensified. In this study, probiotic biofilms of *Lactiplantibacillus plantarum* and *Lacticaseibacillus rhamnosus* were manufactured using milk and transferred to yogurt in whole and pulverized forms to test in real food conditions. Survival was assessed during 21 days of storage time as well as gastrointestinal conditions. The results indicated that *Lp. plantarum* and *Lc. rhamnosus* can form a very desirable and strong biofilm that can have a good protective effect on the survival of these bacteria in probiotic yogurt during processing, storage, and gastrointestinal conditions, in a way that, after 120 min of treatment in high acidic gastrointestinal conditions (pH 2.0), the survival rate decreased by only 0.5 and 1.1 log CFU/ml. Probiotic biofilm can be used as a natural way of utilizing bacteria in biotechnology and fermentation, which is an excellent way to increase the utility of probiotics.

## Graphical abstract

Probiotic biofilms of *Lactiplantibacillus plantarum* and *Lacticaseibacillus rhamnosus* were manufactured using milk. The probiotic biofilms showed excellent gastrointestinal resistance. The probiotic biofilm was used to prepare the dairy product yogurt. The viability of the lactic acid bacteria was greatly increased in highly acidic conditions.

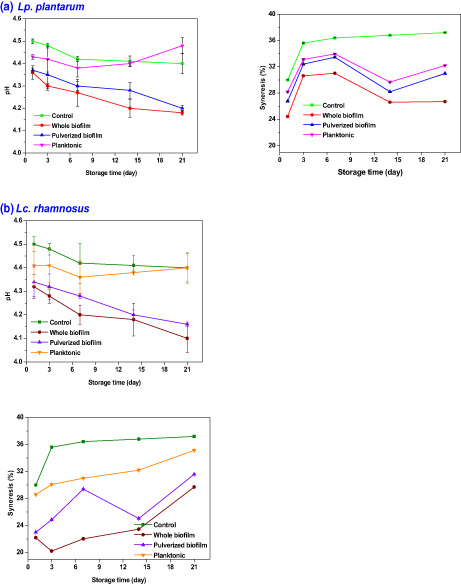

## 1 INTRODUCTION

Probiotics are living microorganisms that, when administered in appropriate amounts, provide health benefits to humans. In recent years, probiotics have seen a significant increase in production due to increased public awareness of their unique properties such as detoxification, cholesterol reduction, normalization of the microbiome, and stimulation of the immune system (Hu et al., 2019). This group of bacteria are useful microorganisms that, by settling in the intestinal environment, can correct the microbial balance to enhance its usefulness (Kaushik et al., 2009; Rezaei et al., 2021). Efforts to produce probiotic products over the past few decades have resulted in the first generation of probiotics. Initially, lyophilized planktonic bacteria were used to produce probiotic dairy products such as yogurt. Later, to address the problem of declining probiotic populations in food processing, storage, and gastrointestinal conditions, the second generation of probiotics was created in which bacteria are coated with natural or synthetic polymers before freeze‐drying (Salas‐Jara et al., 2016). However, this method did not address the sensitivity and vulnerability of the digestive system (Burgain et al., 2011). To solve the problem of second‐generation probiotics, a method of encapsulating probiotics was used, called third‐generation probiotics. This method involves the entrapment of probiotics by mechanical or physicochemical processes such as extrusion, emulsification, coacervation, and spray drying into certain polymeric materials. This method is based on the encapsulation of microbes with nanometer to millimeter‐sized biopolymers to improve probiotic survival and promote controlled release in the gastrointestinal tract (Burgain et al., 2011). However, despite numerous studies on probiotics encapsulation, the problems of probiotics survival and reaching the target site intact have not been fully realized. Therefore, great efforts have been made to commercialize probiotics and introduce them to related industries by developing fourth‐generation probiotics utilizing the unique characteristics of biopolymer films (Cheow & Hadinoto, 2013). Biofilms are complex communities of colonized microorganisms attached to a specific epithelium via an extracellular polysaccharide matrix. This structure is a three‐dimensional network connected by channels, with microbial cells being interconnected (Liu et al., 2015). More than 97% of the composition of this structure is water, which has a tremendous effect on the circulation of nutrients within the biofilm matrix. Other components of the biofilm matrix can include proteins and polysaccharides of around 1% to 2%, as well as DNA and RNA <1% (Lu & Collins, 2007). In biofilms, the bacterium takes a different approach and adapts itself to existing conditions. They also communicate with each other through a mechanism called bacterial quorum sensing (Naves et al., 2010). Antibiotic residues, different processes, and the physicochemical characteristics of the product can reduce the probiotic bacteria (De Vuyst, 2000). The biofilm production strategy can be considered as a useful and effective solution for retrofitting against these factors (Speranza et al., 2020). Therefore, this study aims to achieve probiotic biofilm formation of lactic acid bacteria (*Lacticaseibacillus rhamnosus* and *Lactiplantibacillus plantarum*) in milk, inspired by the detection of a quorum of bacteria in biofilm mode to improve the viability and functional activity of bacteria in probiotic products.

## 2 MATERIALS AND METHODS

### 2.1 Materials

Lyophilized culture of *Lp. plantarum* PTCC 1745 and *Lc. rhamnosus* PTCC 1637 isolated from pickled cabbage was supplied by the Iranian Research Organization for Science and Technology (code: I124). MRS broth and MRS agar (De Man, Rogosa, Sharpe, Merck KGA) were used to culture the bacteria. Fresh milk for yogurt production was obtained from a local agricultural center.

### 2.2 Preparation of lyophilized bacteria

The microbial culture was activated and transferred into De Man, Rogosa, and Sharpe agar (MRS) (Merck KGA). Inoculated plates were incubated for 1–3 days at 37°C under static conditions. The colonies were collected with a sterilized loop and suspended in sterile distilled water. The bacterial suspension was adjusted to (10^8^ CFU/ml) to reach a target inoculum (Kalantarmahdavi et al., 2021).

### 2.3 Preparation of biofilm in culture medium

One milliliter of strains suspension (1.5 × 10^8^ CFU/ml) inoculated with 9 ml of fresh MRS broth was dispensed per well in a 24‐well microplate and incubated at 30°C for 48 h. After incubation, the medium was poured, and the plates were washed twice with sterile distilled water to remove planktonic cells attached to the biofilm. The samples were prepared for imaging (Kubota et al., 2009).

### 2.4 Preparation of biofilm in milk

Milk was used as a substrate precursor. The 6 oz polystyrene straight‐sided jar (2.75 cm diameter × 2.76 cm height) with polypropylene screw cap was used. Two milliliters of strains suspension (1.5 × 10^8^ CFU/ml) inoculated with 18 ml of pasteurized fresh milk (3% fat) and poured into each container and incubated for 48 h at 30°C. After incubation, the excess milk was poured, and samples were washed twice with sterile distilled water.

#### 2.4.1 Characteristics of the biofilm

##### Analysis of composition

One gram of biofilm was used to measure protein and nitrogen by the Kjeldahl method. To measure the content of dry matter, moisture, ash, and polysaccharide, 5 g of biofilm were used and the measurements were performed according to the method by Bradley et al. (1992).

##### Biofilm thickness

Biofilm thickness was measured at 10 random positions using a digital micrometer (Mitutoyo No. 293‐766) with exactness of 1 μm (Kalantarmahdavi et al., 2021).

##### Microstructure of biofilm

Morphology of biofilms in milk and culture medium was observed using a LEO1450VP scanning electron microscope. Biofilm samples were fixed using 2.5% glutaraldehyde for 24 h at 4°C, then washed thrice for 15 min in 10 mM sodium cacodylate buffer by gentle mixing at room temperature, dehydrated in a graded ethanol series 15 min each at 50, 70, 80, 90, and 95, 2 × 15 min at 100% and 3 × 15 min in t‐butyl alcohol, and finally air‐dried at room temperature. After sputter coating with gold, the biofilm sample was observed by SEM with resolution 2.5 nm and Maximum Voltage 35 kV. Images were taken in different magnifications at a voltage of 20 kV (Kubota et al., 2009).

### 2.5 Preparation of probiotic yogurt

Sterile and homogenized bovine milk (fat content of 3%, protein content of 3%, and dry matter content of 8.7%) was heated at 92°C for 12 min and rapidly cooled to 44°C. The direct starter cultures (Micromilk S.R.I.; 2 kg of batch starter/100 kg milk, consisting of *Streptococcus thermophilus* and *Lactobacillus delbrueckii* spp. *bulgaricus*) were added to milk. The thoroughly mixed milk was transferred to containers (150 ml) containing whole biofilm (pure biofilm, the biofilm was pure and without any changed) and pulverized biofilm (the biofilm was completely pulverized and slowly mixed up), respectively. The planktonic cells of *Lp. plantarum* and *Lc. rhamnosus* (1.5 × 10^8^ CFU/ml) were inoculated in another group of samples. All samples were incubated at 37°C, and when the appropriate pH of the yogurt (typically around 4.5) was reached, the samples were stored at refrigerated temperature (4°C) for 3 weeks while monitoring the number of viable bacteria (Yangilar & Yildiz, 2018).

### 2.6 Properties of yogurt

#### 2.6.1 Determination of pH

The pH value of the yogurt was measured using a pH meter (Martini, Mi 151) at regular time intervals (1, 7, 14, and 21 days) during storage at 4°C.

#### 2.6.2 Determination of syneresis

To evaluate the syneresis, 25 g of yogurt was centrifuged at 1500 *g* for 10 min to measure the amount of whey separated, and the syneresis was expressed as a percentage of the amount of whey to the initial amount of yogurt (Domagała, 2009).

#### 2.6.3 Sensory evaluation

Fifteen experienced panelists were used to evaluate yogurt quality. Each panelist was given 40 g of samples stored in the refrigerator with a random code and asked to rinse their mouths with water between evaluations of each sample. Five points hedonic scale (from 1, very dislike to 5, extremely like) was used to evaluate the quality of yogurts such as appearance, texture, taste, and overall acceptance during the storage period of 1, 3, 7, 14, and 21 days (Singh & Muthukumarappan, 2008).

### 2.7 Enumeration of probiotic bacteria in yogurt

After thoroughly mixing, 1 ml of each yogurt sample was diluted with 9 ml of peptone water (0.1%, w/v) to prepare serial dilutions. Appropriate dilutions were plated on set MRS agar containing 10 mg/L of vancomycin and incubated in a plastic anaerobic jar with C type gas pack sachet (Merck KGaA) at 37°C for 48 h. The total number of viable bacteria was expressed as Log CFU/g (Li et al., 2017).

### 2.8 In vitro gastrointestinal tolerance assay

Biofilm of probiotic strains was formed in milk medium and directly tested in simulated stomach and intestine conditions after washing with distilled water. Also, probiotic strains in planktonic form were evaluated as a control. Simulated gastric juice (SGJ) was prepared using potassium chloride (1.12 g/L), sodium chloride (2.0 g/L), calcium chloride (0.11 g/L), and potassium phosphate monobasic (0.4 g/L) after sterilization at 121°C for 15 min. Then, pepsin (0.26 g/L) was added, and the pH was adjusted (~2) by adding 1 N HCl. Then, 1 g of the bacterial biofilms and 3 ml of the suspension containing free cells of lactic acid bacteria were separately added into the containers 30 ml of the gastric juice and digested on a shaker at a rate of 90 rpm at 37°C. At predetermined time intervals (0, 30, 60, 90, and 120 min), the viable bacteria count of solutions was determined by the conventional plate counting method using an MRS agar plate. In the next step, the porcine pancreatin (1.95 g/L) and bovine bile salt (0.18 g/L) (Sigma‐Aldrich) were added to all containers from the previous stage and adjusted the pH to 7.0 using 1 N NaHCO_3_. The containers were kept in the incubator in the shaking condition, and viable bacteria were cultured every hour for 4 h (60, 120, 180, and 240 min). All tests were performed in triplicate (Gebara et al., 2013).

### 2.9 Statistical analysis

All the tests were performed in triplicate or more replications, and results were presented as mean values and the standard deviation. The one‐way analysis of variance (ANOVA) was performed, and significant difference between treatment groups was determined with Duncan's multiple range test at *p* = .05 using the statistical analysis system (SPSS Inc.).

## 3 RESULTS AND DISCUSSION

### 3.1 Biofilm formation on polystyrene containers

**FIGURE 1:**
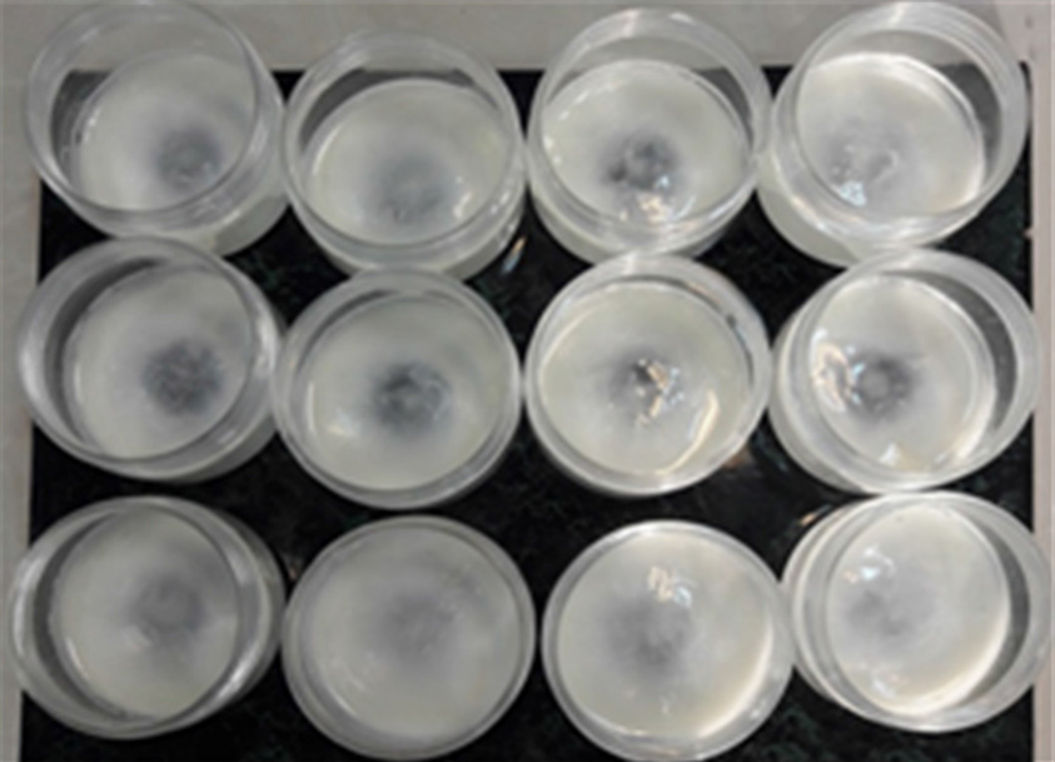
Biofilm formation by probiotic lactic acid bacteria in milk on the surface of PS containers.

Polystyrene containers with 150 volumes were used for biofilm formation. *Lactiplantibacillus plantarum* and *Lc. rhamnosus* cells were attached to the vessel's bottom as fixed support (Figure 1). After 48 h of incubation, a clear biofilm was formed. The biofilm formed interlocks to form a cohesive structure, and the biofilm covers almost all surfaces of the vessel. The lactic acid bacteria in biofilms were tightly associated together. Channels and pores exist in biofilms, which are one of the characteristic structures of biofilms. In these environments, bacterial quorum sensing (QS) plays an important role in controlling biofilm composition and cell number.

### 3.2 Characteristics of the biofilm

#### 3.2.1 Analysis of composition

**FIGURE 2:**
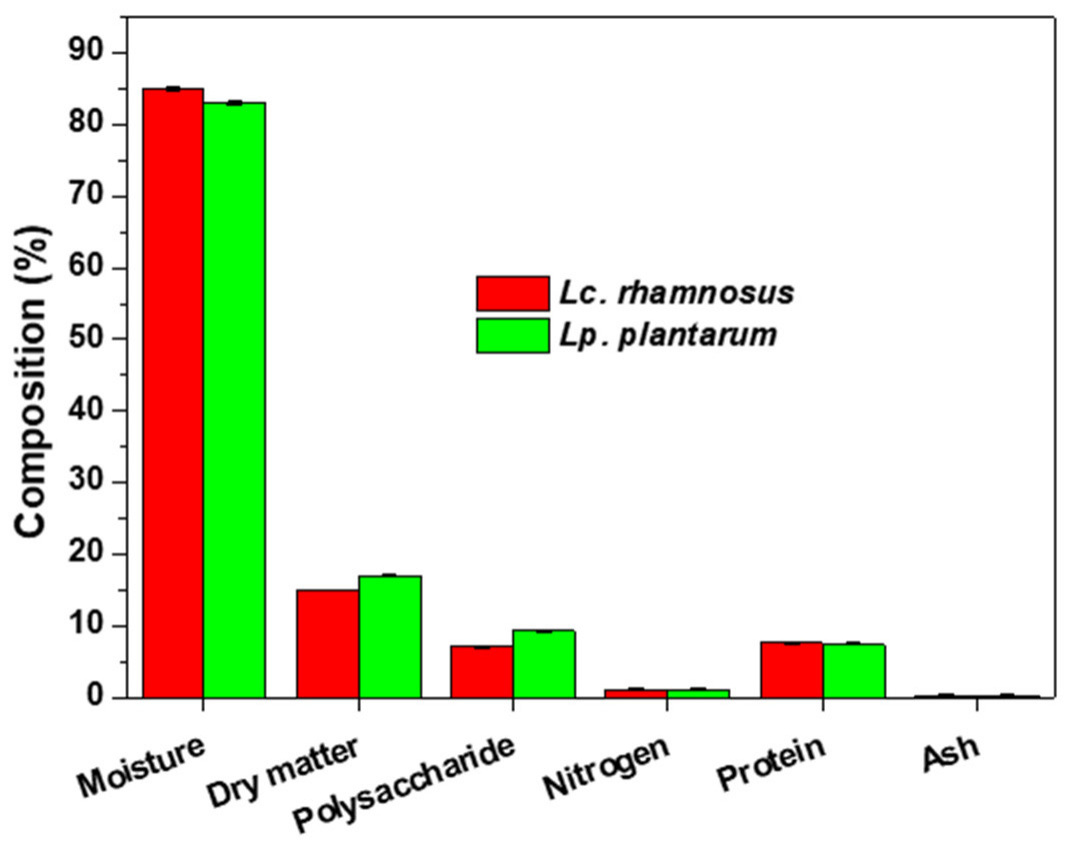
Chemical composition of biofilm of lactic acid bacteria.

The chemical composition of lactic acid bacteria biofilms is shown in Figure 2. Biofilms from probiotic strains contain more protein and polysaccharides and less water than biofilms from pathogenic bacteria, which may be due to the properties of probiotics and their growth media (Dufour et al., 2010). Previous studies have shown that the more proteins and polysaccharides the biofilm contain, the greater the protective effect (Limoli et al., 2015). There was no significant difference (*p* > .05) in the chemical composition and content of the two probiotic strains in this study.

#### 3.2.2 Biofilm thickness

The thickness of biofilms of *Lc. rhamnosus* and *Lp. plantarum* was 280 ± 25 and 300 ± 20 μm, respectively. There was no significant difference in biofilm thickness produced by the two strains (*p* < .05). However, some previous studies have reported that the biofilm production capacity depends on the type of strain, and intraspecies differences in biofilm production have been reported (Ramírez et al., 2015).

#### 3.2.3 Microstructure of biofilm

**FIGURE 3:**
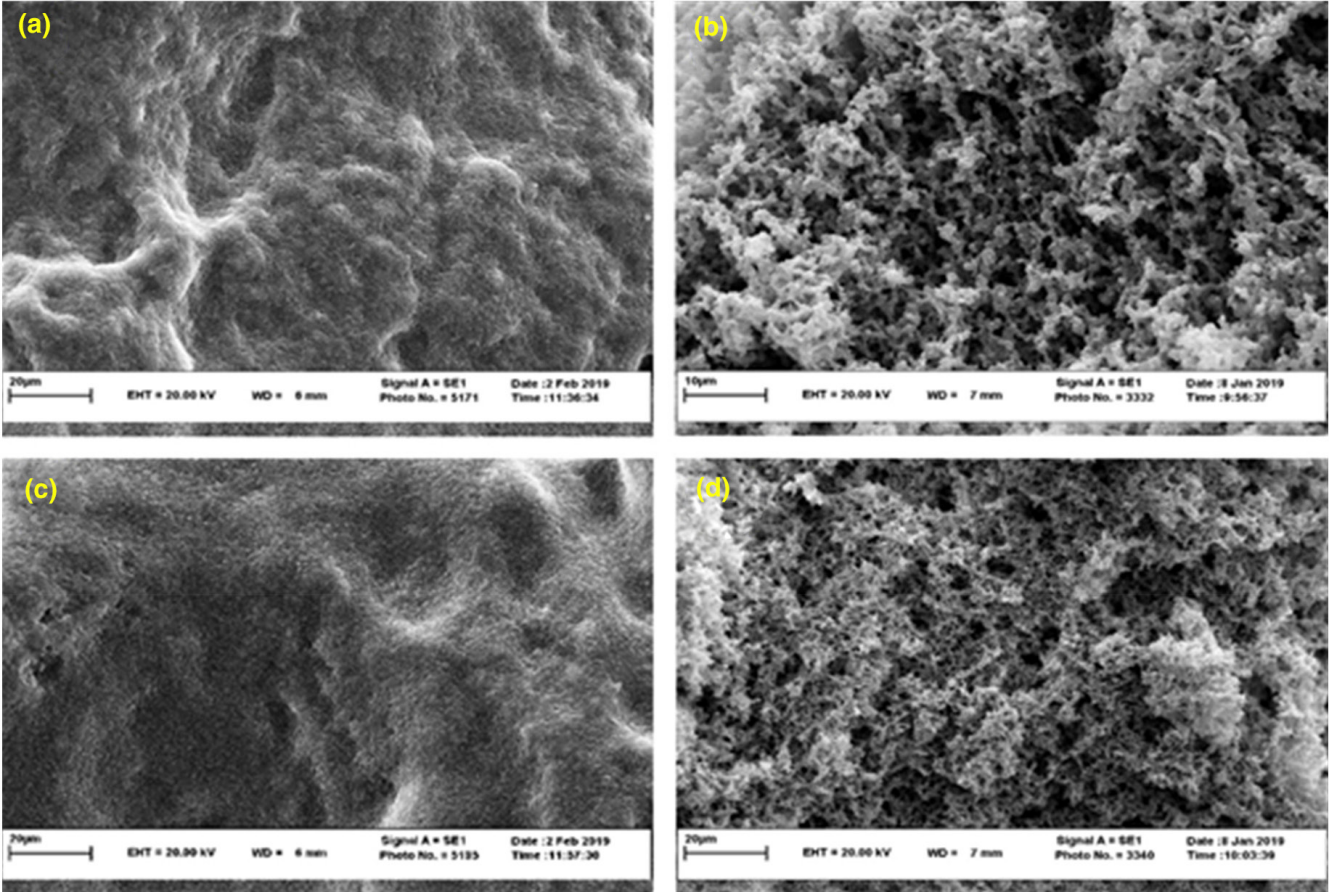
Scanning electron microscopy images: biofilm of *Lacticaseibacillus rhamnosus* in MRS agar (a), biofilm of *Lc. rhamnosus* in milk (b), biofilm of *Lactiplantibacillus plantarum* in MRS agar and (c), biofilm of *Lp. plantarum* in milk (d).

The biofilm microstructure in MRS broth and milk medium of the lactic acid bacteria (*Lc. rhamnosus* and *Lp. plantarum*) was observed using SEM (Figure 3). *Lacticaseibacillus rhamnosus* biofilms grown in milk showed a complex three‐dimensional structure, and diffuse extracellular material was observed due to the aggregation of bacterial cells. By contrast, the biofilms in the MRS broth culture medium showed a less dense structure. *Lactiplantibacillus plantarum* also formed a stronger biofilm in milk than MRS broth. All biofilm structure studies were performed in culture media (Jones & Versalovic, 2009). So far, there have been no studies on biofilm formation using food media. The mechanism of this phenomenon is not discovered, yet it needs more genetically and structural works.

### 3.3 Viability of probiotics in yogurt during storage

**FIGURE 4:**
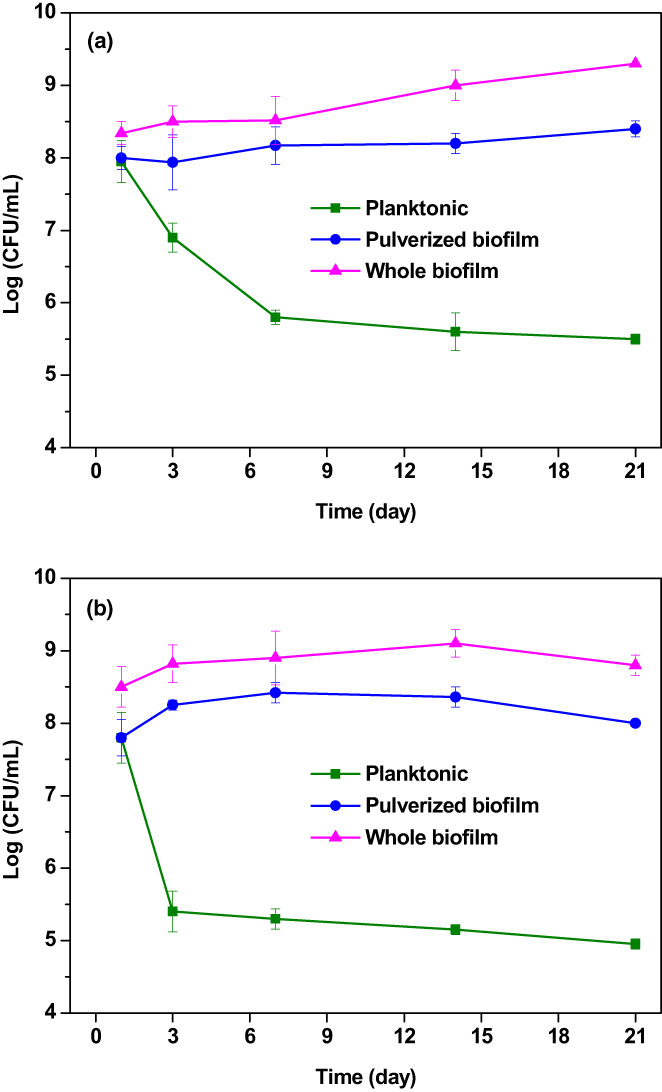
Viability of (a) *Lacticaseibacillus rhamnosus* and (b) *Lactiplantibacillus plantarum* during storage for 21 days at 4°C. (Control: The form of planktonic).

The viability of *Lp. plantarum* and *Lc. rhamnosus* in the biofilm was evaluated during storage at 4°C, and the results are shown in Figure 4. As expected, the biofilm protected the bacteria early in storage, and the bacterial population was not significantly decreased (*p* > .05). On the contrary, an average decrease of 3.5 Log CFU/ml in the planktonic form was observed. However, the pulverized and whole biofilm showed similar protective effects, and there was no significant difference between them (*p* > .05). These results indicate that if the biofilm structure is well‐formed, each biofilm fragment behaves like the original complete structure and repairs itself. Applying this idea to industrial and probiotic products is paramount because the pulverized probiotic biofilm can be used in products with various formulations and uniform texture, such as pulverized probiotic biofilm and yogurt. However, more studies are needed to understand how to repair mechanisms and stability work in fragments of broken biofilms. As a result of examining the survival process of the bacteria during storage for 3 weeks, it can be seen that the bacteria in the biofilm reduced the metabolism to the basal metabolic rate and were well adapted to the new environment (yogurt) different from the initial environment where it was grown (milk). There was no significant decrease in bacteria during storage as the biofilm could protect the probiotic bacteria well in the new environment. However, a significant decrease (2.83 Log CFU/ml) in planktonic bacteria was observed under similar conditions. The biofilm structure of the bacteria allows it to continue to grow and multiply exponentially in the yogurt environment without being affected by new environmental changes. This good feature can ensure the viability of probiotics at the minimum standard value of 10^6^ in probiotic products. On the contrary, the survival rates of the two bacteria are similar, indicating the general behavior of the biofilm‐forming bacteria of lactic acid bacteria species (Terraf et al., 2012). A recent study of survival using the new method showed a noticeable reduction in bacteria, with reductions observed at various conditions ranging from 1 to 7 Log CFU/ml. However, compared with other common techniques, the biofilm saber‐rattling can be explained by a 3.1 Log CFU/ml increase in the planktonic state, demonstrating the power of the biofilm (Afzaal et al., 2019). Biofilm formation methods could potentially revolutionize the probiotic industry as they are resistant and increase survival in difficult and new conditions (Okuda et al., 2018).

### 3.4 Syneresis and pH of yogurt

**FIGURE 5:**
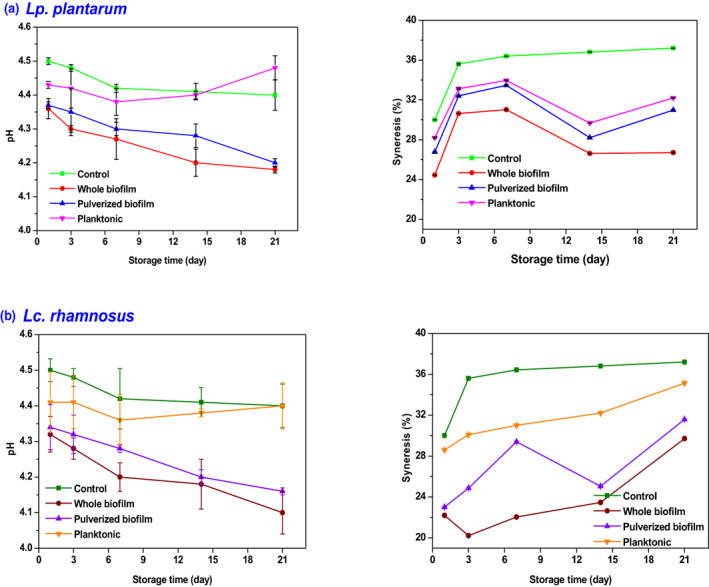
Physicochemical properties of probiotic yogurt with (a) *Lactiplantibacillus plantarum* and (b) *Lacticaseibacillus rhamnosus*.

The syneresis and pH measurement results in yogurt samples containing planktonic cell, whole, and pulverized biofilm of *Lp. plantarum*, *Lc. rhamnosus*, and control samples are shown in Figure 5. The pH of yogurt, including both whole biofilm and pulverized biofilm, has a direct relationship with the survival of the bacteria. Yogurt samples containing pulverized biofilm and whole biofilm have a lower pH than yogurt samples containing the planktonic form of probiotics, probably due to the biofilm's protective effect on probiotics. Because the biofilm can lead to the survival of most probiotics, the bacterial population of more probiotics is present, resulting in higher lactic acid production and lower final pH of the product compared with yogurt samples containing planktonic forms probiotics. Moreover, samples containing whole biofilms had lower pH than samples containing pulverized biofilms, possibly because bacterial populations are better preserved in whole biofilms than in pulverized biofilms (Koohestani et al., 2018). Syneresis is expressed as the amount of exudate moisture in the yogurt accumulated on the yogurt surface. This parameter affects the appearance quality of the yogurt and general product acceptance. To reduce or prevent syneresis, the dairy industry uses stabilizers such as pectin and gum or increases the protein content (Lee & Lucey, 2010). The percentage of syneresis was lower in the yogurt samples containing whole and pulverized biofilm than in the planktonic form due to the biofilm's three‐dimensional structure. Leccese et al. (2016) evaluated the biofilm matrix formed by *Lc. rhamnosus* CRL 1332, showing that the biofilm matrix contains large amounts of polysaccharides, carbohydrates, and proteins (Terraf et al., 2012). These natural compounds produced by probiotic bacteria in the biofilm network can play a similar role to stabilizers. Due to their hydrophilic groups, they can absorb yogurt water. The biofilm integrated structure is an important factor in holding water capacity in the biofilm structure (Salas‐Jara et al., 2016). The channels in the biofilm structure created by water can effectively maintain and absorb water while providing the nutritional requirements of microorganisms. Thus, this is another structural advantage of fourth‐generation probiotics over first‐generation probiotics.

### 3.5 Sensory evaluation of yogurt

The findings of the sensor assessment for the yogurt make with the probiotic biofilm are shown in Table 1. Yogurt containing biofilms was significantly superior color and odor qualities. The acidity of yogurt can affect its pleasant odor. Therefore, the color of the yogurt appears whiter due to the polysaccharide and protein compounds in the sample prepared from the biofilm. On the contrary, the more solids in the product, the more vivid and white the color due to light scattering (Singh & Muthukumarappan, 2008). The texture of yogurt is considered one of the most important parameters. The yogurt prepared with biofilm contains internal force with many bonds due to the biofilm's special and unique structure, resulting in more acceptable than control samples (*p* < .05).

**TABLE 1:** Sensory evaluation of probiotic yogurt made by planktonic and biofilm forms of probiotics.

Sensory parameter	Treatment	Storage time (days)				
		0	3	7	14	21
Appearance	Whole biofilm (*Lp. plantarum*)	3.00 ± 0.93_a_^ab^	2.73 ± 0.70_a_^b^	3.00 ± 0.93_a_^ab^	3.53 ± 0.64_a_^a^	3.07 ± 1.10_a_^ab^
	Pulverized biofilm (*Lp. plantarum*)	3.00 ± 0.76_a_^a^	2.40 ± 1.06_ab_^a^	2.53 ± 1.13_a_^a^	2.40 ± 1.24_c_^a^	2.33 ± 0.98_b_^a^
	Planktonic (*Lp. plantarum*)	2.73 ± 0.96_ab_^a^	2.60 ± 1.06_ab_^a^	2.60 ± 1.12_a_^a^	2.47 ± 0.92_bc_^a^	2.33 ± 1.05_b_^a^
	Whole biofilm (*Lc. rhamnosus*)	3.13 ± 0.74_a_^a^	3.07 ± 0.80_a_^a^	3.00 ± 1.07_a_^a^	3.13 ± 0.74_ab_^a^	3.40 ± 0.74_a_^a^
	Pulverized biofilm (*Lc. rhamnosus*)	3.40 ± 0.74_a_^a^	2.93 ± 0.96_a_^a^	2.87 ± 0.74_a_^a^	3.13 ± 1.06_ab_^a^	3.20 ± 0.86_a_^a^
	Planktonic (*Lc. rhamnosus*)	2.27 ± 1.22_bc_^a^	2.60 ± 0.91_ab_^a^	2.27 ± 1.16_ab_^a^	2.87 ± 0.92_abc_^a^	2.33 ± 0.62_b_^a^
	Control	1.93 ± 1 .03 _c_^a^	1.93 ± 1.03_b_^a^	1.73 ± 0.59_b_^a^	1.60 ± 0.51_d_^a^	1.00 ± 0.00_C_^b^
Texture	Whole biofilm (*Lp. plantarum*)	3.38 ± 0.73_a_^a^	2.90 ± 1.00 _a_^a^	3.00 ± 0.80_ab_^a^	3.33 ± 0.60_a_^a^	3.22 ± 0.86_a_^a^
	Pulverized biofilm (*Lp. plantarum*)	2.85 ± 0.90_a_^a^	3.02 ± 0.95_a_^a^	2.25 ± 1.01_c_^a^	2.94 ± 1.02_a_^a^	2.94 ± 0.72_a_^a^
	Planktonic (*Lp. plantarum*)	2.81 ± 0.84_a_^a^	2.84 ± 1.10_a_^a^	2.61 ± 1.13_bc_^a^	3.06 ± 0.85_a_^a^	2.94 ± 0.87_a_^a^
	Whole biofilm (*Lc. rhamnosus*)	3.10 ± 0.81_a_^a^	2.91 ± 1.01_a_^a^	3.34 ± 0.94_a_^a^	3.42 ± 0.76_a_^a^	3.22 ± 0.77 _a_^a^
	Pulverized biofilm (*Lc. rhamnosus*)	3.09 ± 1.07_a_^a^	3.04 ± 1.22_a_^a^	3.21 ± 0.82_ab_^a^	3.24 ± 0.81_a_^a^	3.08 ± 0.81_a_^a^
	Planktonic (*Lc. rhamnosus*)	1.89 ± 1.20_b_^c^	2.29 ± 1.12_ab_^bc^	2.07 ± 1.10_c_^bc^	3.24 ± 0.81_a_^a^	2.74 ± 0.81_a_^ab^
	Control	1.86 ± 1.23_b_^a^	1.74 ± 1.05_b_^a^	1.01 ± 0.01_d_^b^	1.01 ± 0.01_b_^b^	1.01 ± 0.01_b_^b^
Taste	Whole biofilm (*Lp. plantarum*)	3.33 ± 0.82_a_^ab^	3.13 ± 0.83_a_^b^	3.13 ± 0.74_ab_^ab^	3.20 ± 0.68_a_^a^	3.13 ± 0.84_a_^ab^
	Pulverized biofilm (*Lp. plantarum*)	3.00 ± 0.66_a_^ab^	2.80 ± 0.86_ab_^a^	2.47 ± 0.99_bc_^ab^	2.40 ± 0.74_b_^ab^	2.07 ± 0.59_b_^b^
	Planktonic (*Lp. plantarum*)	3.00 ± 1.00_ab_^a^	3.07 ± 0.88_ab_^a^	2.60 ± 0.83_abc_^a^	2.80 ± 0.78_ab_^a^	2.60 ± 0.91_ab_^a^
	Whole biofilm (*Lc. rhamnosus*)	2.93 ± 0.88_ab_^a^	2.87 ± 0.83_ab_^a^	3.20 ± 0.94_a_^a^	3.33 ± 0.62_a_^a^	3.07 ± 0.59 _a_^a^
	Pulverized biofilm (*Lc. rhamnosus*)	3.00 ± 1.00_ab_^a^	3.07 ± 1.16_ab_^a^	3.13 ± 0.83_ab_^a^	3.33 ± 0.82_a_^a^	3.00 ± 0.76_a_^a^
	Planktonic (*Lc. rhamnosus*)	2.07 ± 1.22_c_^b^	2.40 ± 0.83_bc_^ab^	2.13 ± 1.06_c_^ab^	2.93 ± 0.80_ab_^a^	2.27 ± 1.16_b_^ab^
	Control	2.27 ± 1.22_bc_^a^	2.07 ± 1.05_c_^a^	1.47 ± 0.52_d_^b^	1.33 ± 0.49_c_^b^	1.00 ± 0.00_C_^b^
Overall acceptance	Whole biofilm (*Lp. plantarum*)	3.40 ± 0.74_a_^a^	2.93 ± 1.03_a_^a^	3.07 ± 0.88_ab_^a^	3.40 ± 0.63_a_^a^	3.27 ± 0.88_a_^a^
	Pulverized biofilm (*Lp. plantarum*)	2.87 ± 0.92_a_^a^	3.00 ± 0.93_a_^a^	2.27 ± 1.03_c_^a^	2.93 ± 1.03_a_^a^	2.93 ± 0.70_a_^a^
	Planktonic (*Lp. plantarum*)	2.80 ± 0.86_a_^a^	2.87 ± 1.13_a_^a^	2.60 ± 1.12_bc_^a^	3.07 ± 0.88_a_^a^	2.93 ± 0.88_a_^a^
	Whole biofilm (*Lc. rhamnosus*)	3.13 ± 0.83_a_^a^	2.93 ± 1.03_a_^a^	3.33 ± 0.98_a_^a^	3.40 ± 0.74_a_^a^	3.20 ± 0.76 _a_^a^
	Pulverized biofilm (*Lc. rhamnosus*)	3.07 ± 1.10_a_^a^	3.00 ± 1.20_a_^a^	3.20 ± 0.86_ab_^a^	3.27 ± 0.80_a_^a^	3.07 ± 0.80_a_^a^
	Planktonic (*Lc. rhamnosus*)	1.87 ± 1.30_b_^c^	2.27 ± 1.10_ab_^bc^	2.07 ± 1.10_c_^bc^	3.27 ± 0.80_a_^a^	2.73 ± 0.80_a_^ab^
	Control	1.87 ± 1.25_b_^a^	1.73 ± 1.03_b_^a^	1.00 ± 0.00_d_^b^	1.00 ± 0.00_b_^b^	1.00 ± 0.00_b_^b^

*Note*: Index letters and power letters indicate the comparison of the averages in the columns and rows, respectively (*p* ≤ .05).

On the contrary, extracellular polysaccharides in the biofilm have led to a perfect mouth feeling. The present results showed that yogurt samples prepared with the biofilm taste significantly better than the control samples, and this property was maintained during the storage period. Since the biofilm matrix contains around 6%–7% protein and polysaccharides, these compounds may affect other tissue properties of the product (Mousavi et al., 2019). In addition, the biofilm has the effect of protecting the number of bacteria in the probiotics, which increases the number of bacteria and lactic acid production, and improves the acidity and taste of the product. The yogurt prepared with the biofilms showed significantly (*p* < .05) higher sensory values in all cases compared with control and planktonic samples (without biofilm). These results indicate that the new yogurt has a higher industrial potential and higher overall acceptability scores in sensory evaluation. Comparing the results with other techniques, such as nanoencapsulation and microencapsulation (Yao et al., 2020), showed that biofilms did not cause negative sensory changes but could also be used to improve the organoleptic properties of yogurt and similar products.

### 3.6 Viability in gastrointestinal simulation condition

**FIGURE 6:**
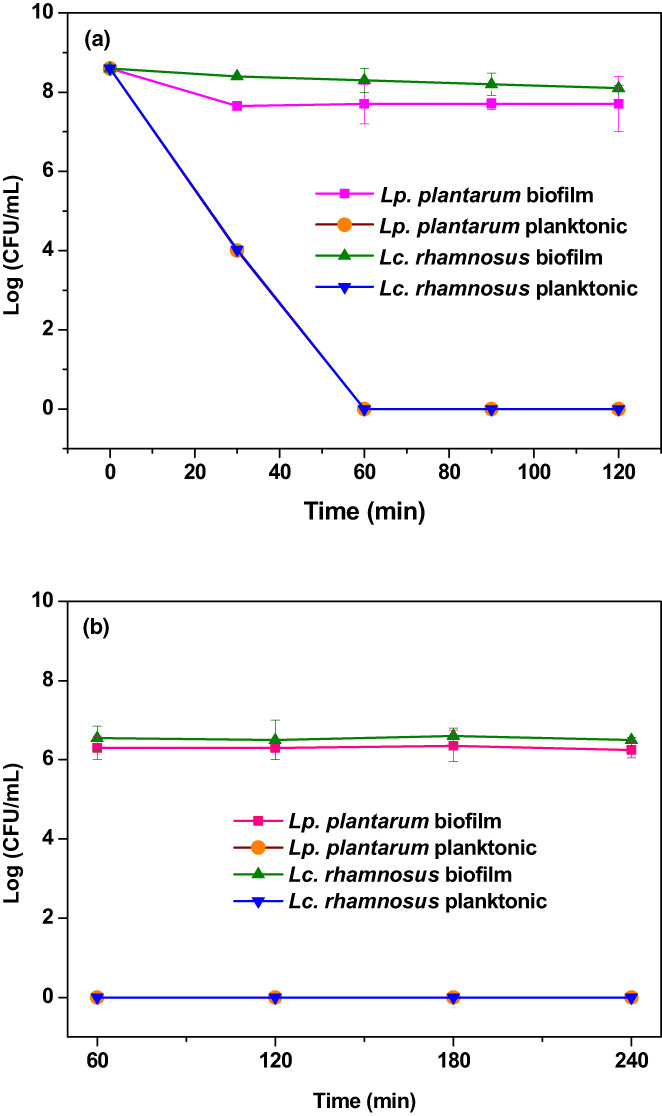
Viability of lactic acid bacteria biofilm during the exposure to (a) the simulated gastric condition (pH 3.0) and (b) the simulated intestine condition (pH 7.0).

Cell viability is a very important indicator in assessing the gastrointestinal resistance of probiotics. The viability of the probiotic bacteria biofilms in simulated gastrointestinal conditions shows the gastrointestinal resistance of probiotic bacteria, which can survive in the acidic and alkalinity environment and then reach the targeted regions of the intestine. Planktonic form of strains was used as the control in this test. As shown in Figure 6, the viability of probiotic bacteria in the biofilm and planktonic form during sequential exposure simulated gastric juice (SGJ, pH 3.0) for 120 min and simulated intestinal juice (SIJ, pH 7.0) for 240 min. In gastric conditions, the free cell viability of *Lc. rhamnosus* and *Lp. plantarum* was decreased to 4.03 and 4 Log CFU/ml after 30 min, respectively, and decreased to undetectable levels after 60 min.

In comparison, the viability of *Lc. rhamnosus* and *Lp. plantarum* was greatly enhanced by biofilm in the high acid condition (pH 3.0) to the extent that only 0.5 and 1.1 Log CFU/ml reduction was observed in 120 min and maintained at 8.10 and 7.70 Log CFU/ml, respectively. After gastrointestinal digestion analysis, it can be concluded that the biofilm method positively affects the viability of probiotic cells. Comparing the results of the new biofilm method with the third‐generation probiotics (encapsulation) revealed that even in relatively advanced protection techniques such as nanocomposites and microencapsulation showed a 1–7 logarithmic reduction in the number of bacteria, but the biofilm, a unique natural method, showed an amazing performance in increasing the survival of probiotics (Afzaal et al., 2019). A unique feature of this new method is its naturalness, a characteristic inherent in bacteria. Sohail et al. (2011) reported that encapsulation of probiotics in alginate gel microbeads could protect probiotics in a highly acidic environment, but with a greater viability reduction than the biofilm method (Sohail et al., 2011). Huq et al. (2017) also reported that the viability of the probiotic bacterium *Lc. rhamnosus* in alginate‐based nanocomposites was reduced by 1.45 Log CFU/ml after 120 min in a simulated gastric environment (Huq et al., 2017). The present results have shown that biofilms have higher efficiency in the survival of *Lc. rhamnosus*. Viability studies in simulated intestinal environments have also shown interesting results. As shown in Figure 6, the test probiotics slightly decreased during the movement from the stomach to the intestine, but increased the next time after digestion. Finally, after 4 h of incubation, the final reductions for primary cells for *Lc. rhamnosus* and *Lp. plantarum* were 0.59 and 1.05 Log CFU/ml, respectively. González‐Ferrero et al. (2018) found that the reduction rates of *Lp. plantarum* and *L. casei* in the encapsulated state after incubation time were 1 and 1.4 Log CFU/ml, respectively, in good agreement with the results (González‐Ferrero et al., 2018). However, *Lc. rhamnosus* biofilm showed a higher survival rate than *Lp. plantarum* biofilm. This is consistent with previous studies that the strength of biofilm formation depends on the bacterial strain (Rezaei et al., 2021). Liao et al. (2019) reported that *Li. fermentum* reduced approximately 1 Log CFU/ml in the encapsulated state after 240 min of incubation (Liao et al., 2019). Pop et al. (2017) also reported the viability of *L. casei* was reduced by about 1 Log CFU/ml after 120 min of incubation (Pop et al., 2017). The present findings demonstrated the unique ability of the biofilm technique to maintain the viability of probiotics in simulated gastric and intestinal conditions, which may be cheaper, simpler, and more efficient than the third generation of probiotics.

## 4 CONCLUSIONS

Probiotic biofilms of lactic acid bacteria, *Lc. rhamnosus* and *Lp. plantarum*, were prepared using milk as a food medium and used to prepare yogurt. The probiotic biofilms can grow well on food media and prepare probiotic products. The probiotics in the biofilm can withstand gastrointestinal conditions and are expected to enter the intestines without significant destruction. Food‐based probiotic bacteria biofilms is a safe and efficient way to use probiotics and is expected to have a high potential for use in food processing, biotechnology, and fermentation engineering industries.

## CONFLICT OF INTEREST STATEMENT

The authors declare no conflict of interests.

## Data Availability

Data are available on request from the authors.
